# Muscle activities during walking and running at energetically optimal transition speed under normobaric hypoxia on gradient slopes

**DOI:** 10.1371/journal.pone.0173816

**Published:** 2017-03-16

**Authors:** Daijiro Abe, Yoshiyuki Fukuoka, Masahiro Horiuchi

**Affiliations:** 1 Center for Health and Sports Science, Kyushu Sangyo University, Fukuoka, Japan; 2 Faculty of Health and Sports Science, Doshisha University, Kyotanabe, Japan; 3 Division of Human Environmental Science, Mt. Fuji Research Institute, Fujiyoshida, Japan; University of France Comté, FRANCE

## Abstract

Energy cost of transport per unit distance (CoT; J·kg^-1^·km^-1^) displays a U-shaped fashion in walking and a linear fashion in running as a function of gait speed (*v*; km·h^-1^). There exists an intersection between U-shaped and linear CoT-*v* relationships, being termed energetically optimal transition speed (EOTS; km·h^-1^). Combined effects of gradient and moderate normobaric hypoxia (15.0% O_2_) were investigated when walking and running at the EOTS in fifteen young males. The CoT values were determined at eight walking speeds (2.4–7.3 km·h^-1^) and four running speeds (7.3–9.4 km·h^-1^) on level and gradient slopes (±5%) at normoxia and hypoxia. Since an alteration of *tibialis anterior* (TA) activity has been known as a trigger for gait transition, electromyogram was recorded from TA and its antagonists (*gastrocnemius medialis* (GM) and *gastrocnemius lateralis* (GL)) for about 30 steps during walking and running corresponding to the individual EOTS in each experimental condition. Mean power frequency (MPF; Hz) of each muscle was quantified to evaluate alterations of muscle fiber recruitment pattern. The EOTS was not significantly different between normoxia and hypoxia on any slopes (ranging from 7.412 to 7.679 km·h^-1^ at normoxia and 7.516 to 7.678 km·h^-1^ at hypoxia) due to upward shifts (enhanced metabolic rate) of both U-shaped and linear CoT-*v* relationships at hypoxia. GM, but not GL, activated more when switching from walking to running on level and gentle downhill slopes. Significant decreases in the muscular activity and/or MPF were observed only in the TA when switching the gait pattern. Taken together, the EOTS was not slowed by moderate hypoxia in the population of this study. Muscular activities of lower leg extremities and those muscle fiber recruitment patterns are dependent on the gradient when walking and running at the EOTS.

## Introduction

**Fig 1:**
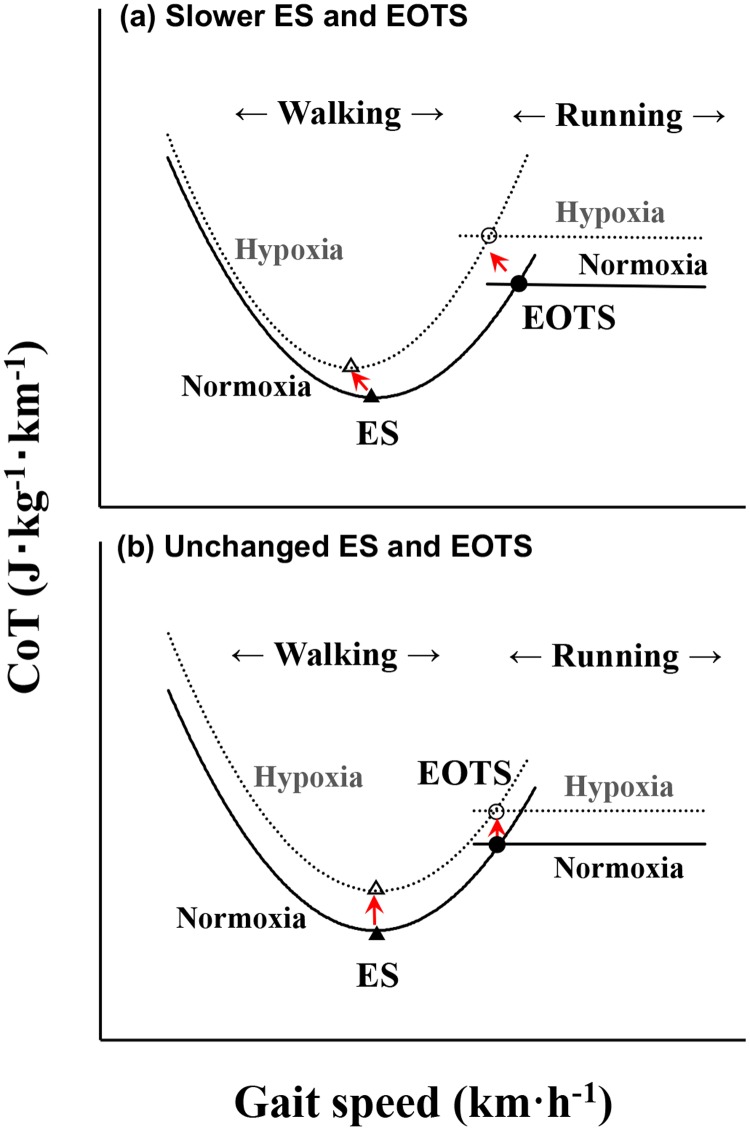
Schematic illustration of Cost of Transport (CoT) and gait speed (*v*) at normoxia and hypoxia. (a) Upward and leftward shifts of the U-shaped CoT-*v* relationship in walking and an upward shift of the linear CoT-*v* relationship in running result in a slower energetically optimal transition speed (EOTS, circles) and economical speed (ES, triangles) at hypoxia. (b) Upward shifts of both CoT-*v* relationships result in unchanged ES and EOTS. Solid and dotted lines mean normoxia and hypoxia, respectively. Both red arrows indicate a possible ‘shifting model’ for explaining slower ES and EOTS at hypoxia.

Human land locomotion is characterized by erect bipedalism, and its biological benefit has been known to be economical in walking [1,2] and running [1,3,4]. There is a U-shaped relationship between energy cost of transport per unit distance (CoT; J·kg^-1^·km^-1^) and gait speed (*v*; km·h^-1^) during walking [3–5] and a linear relationship during running [5–7]. Thus, there is an intersection between U-shaped and linear CoT-*v* relationships, being termed ‘energetically optimal transition speed’ (EOTS; km·h^-1^) [6–10]. There also exists a specific walking speed that can minimize the CoT in each individual. Such a specific walking speed has been known as economical speed (ES; km·h^-1^) [7,11–14]. It is interesting to note that the EOTS and ES has not been reported on gradient slopes under hypoxic condition, although it is reported that more than 6.3 billion people are living at elevations above 2500 m in the world [15]. Ventilation increases when exposed to an acute normobaric or hypobaric hypoxia [16,17], suggesting that a possible increase in the ventilatory cost may affect the U-shaped and linear CoT-*v* relationships higher when exposed to hypoxic condition (Fig 1a or 1b).

The metabolic minimization during locomotion is an important biological optimization, so that the EOTS is metabolically related to a gait change from walking to running in bipedal locomotion [18–20]. The EOTS appeared around 7–8 km·h^-1^ on the level slope in adult males, being close to preferred gait transition speed (PTS) [6,8–10,18]. Several biomechanical studies showed that an abrupt increase in the muscle activities of lower limbs measured by electromyogram (EMG), particularly in *tibialis anterior* (TA), is a potential trigger for walk-to-run gait change [21–27]. Here, it is questionable whether an elevated TA activity is the only trigger for the gait change, because TA mainly activates during swing phase, but not during push-off phase [21]. Planter flexors are rather associated to produce mechanical power for the propulsion [28]. Muscle fatigue will be facilitated at hypoxia mainly at faster walking speeds. When exercising muscles fatigue, greater muscle activation must be generated to maintain the same level or intensity of the performance. If elevated muscle activity at faster walking speeds is the trigger to start running, then an increased muscle recruitment will also result in a slower gait change speed at hypoxia. Different gradient slopes will emphasize these possibilities, because these influence on both metabolic responses and mechanical stress on the lower extremities [29].

Under consideration of muscle activities and metabolic profiles, a combination of different gradient slopes and inspired oxygen levels will be available for understanding the link between metabolic minimization during human bipedal locomotion and muscular activities in the lower extremities at the EOTS. It is hypothesized that the EOTS and ES will be slower at hypoxia than normoxia on any slopes. The second hypothesis was established that the TA activity will be decreased in association with an alteration of its muscle fiber recruitment when switching from walking to running at the EOTS. It was further hypothesized that the ES and EOTS will be faster in order of downhill, level and uphill slope under each oxygen conditions. To test these hypotheses, this study quantified the U-shaped and linear CoT-*v* relationships at normobaric hypoxia (15.0% O_2_, equivalent altitude is about 2700 m) and normoxia (20.9% O_2_) on the level and gradient slopes to examine combined effects of hypoxia and gradient on the EOTS and shank muscle activities during walking and running corresponding to the EOTS.

## Methods

### Participants

Fifteen physically active males, who did not have a cardiorespiratory sickness and musculoskeletal disorders, participated in this study. The mean age, height, and body mass were 20.7 ± 1.2 years old, 1.71 ± 0.06 m, and 62.6 ± 4.4 kg, respectively (mean ± standard deviation; SD). A written informed consent was obtained from all participants after medical check and detailed explanations of all procedures, purpose of this study, possible risks, and benefits of the participation. This study conformed the Declaration of Helsinki, and an ethical committee established in Kyushu Sangyo University approved all procedures of this study (H27-0002).

### Experimental protocols

All studies were carried out on a motor-driven treadmill (LABORDO LXE1200, Senoh, Japan). The participants wore underwear, shirts, socks, shorts and same training shoes (Wave Wing, Mizuno, Japan) with different sizes. The treadmill slopes were set at 0% (level), -5% (downhill), and +5% (uphill) [7,12,13]. These different slopes are expected to modify not only metabolic rate but also each muscle activity. Inspired oxygen fraction (FiO_2_; %) was set at normobaric normoxia (20.9% FiO_2_) and hypoxia (15.0% FiO_2_). Experimental conditions were summarized in Table 1. Fifteen percent of FiO_2_ is equivalent to the altitude about 2700 m. The order of slope and FiO_2_ was randomized, and the participants performed one of these experimental measurements once in one experimental day. That is, each participant completed 7 experimental measurements on separate days (6 metabolic and 1 EMG measurements). On each measurement, the participants walked and ran on the treadmill with a freely chosen step frequency at several gait speeds. Eight gait speeds were incrementally set at 2.4, 3.1, 3.8, 4.5, 5.2, 5.9, 6.6, and 7.3 km·h^-1^ for walking and four gait speeds at 7.3, 8.0, 8.7, and 9.4 km·h^-1^ for running [7]. Running speeds were limited until 9.4 km·h^-1^, because our present study involved uphill slope at hypoxic conditions. The metabolic data were continuously measured during 8 walking speeds, and 1-min rest was inserted among each running stage. These multiple gait speeds tested should be available for a trust worthy approximation of the U-shaped and linear CoT-*v* relationships, which would result in a reliable evaluation for the individual EOTS and/or ES. Between walking and running, the participants sat on a chair to take a rest for 7–8 minutes. S_p_O_2_ was also measured with a pulse oximeter (PULSOX-1, Konica Minolta, Tokyo, Japan) from right index finger at the final minute of each stage.

**Table 1:** Summary of experimental conditions.

Inspired Oxygen		Gradient			Classification
20.9% (Normoxia)		Level (0%)	Uphill (+5%)	Downhill (-5%)	Metabolic measurement
15.0% (Hypoxia)		Level (0%)	Uphill (+5%)	Downhill (-5%)	
20.90%	15.00%	EMG recording (3 Normoxic conditions → 3 Hypoxic conditions)			EMG measurement

Each participant performed one of 6 metabolic measurements on separate days. After all metabolic measurements, electromyogram (EMG) recordings were conducted in another day.

The pulmonary oxygen uptake (*V*O_2_; mL·kg^-1^·min^-1^) was measured with a computerized breath-by-breath system (AE-310S, Minato Medical Science, CO., Ltd, Osaka, Japan). The standard known gases (O_2_ 15.22%, CO_2_ 5.17%, and N_2_ 79.61%) and room air were used for the calibration of gas analyzer. Each gait speed was kept for 4-min, and a single sample of an average *V*O_2_ and carbon dioxide output (*V*CO_2_; mL·kg^-1^·min^-1^) for the final 2-min at each gait speed was used to calculate the CoT using following equation [30,31]:

$$\text{CoT }\left(\text{J}\cdot {\text{kg}}^{-1}\cdot {\text{km}}^{-1}\right)\;=\;\;\frac{4.186\;\times \;1000\;\times \;\left(3.869\times VO2\;+\;1.195\times VCO2\right)}{v}.$$ (1)

The CoT values were compared at each gait speed among different FiO_2_ conditions and slopes to evaluate whether the U-shaped and/or linear CoT-*v* relationships shifted upward.

In human walking, a relationship between CoT and gait speeds can be approximated with a quadratic equation [7,12–14,32,33], and it is described as follows:

$$\text{CoT }\left(v\right)\;=\;av^{2}\;+\;bv\;+\;c.$$ (2)

Where the coefficients *a*, *b*, and *c* are determined by the least squares regression with data obtained from eight walking speeds. The most economical walking speed (ES; km·h^-1^), which minimizes the CoT-*v* relationship in walking, can be obtained when a differential function of the Eq 2 (CoT’ (*v*) = 2*av* + *b*) is zero [7,12–14]. Then the individual ES was determined using following equation, and it was available to evaluate whether the U-shaped CoT-*v* relationship shifted leftward (slower) [12–14]:

$$\text{ES }\left(\text{km}\cdot {\text{h}}^{-1}\right)\;=\;\frac{\left|-b\right|}{2a}$$ (3)

In human running, a relationship between CoT and gait speeds was approximated using a quadratic equation [4,34]. However, running speeds were limited from 7.3 to 9.4 km·h^-1^ as explained before, so that we have four running speeds only. Thus, a linear regression analysis was applied on the running CoT-*v* relationships [7]. The CoT during running can be described as follows:

CoT (v) = pv + q. (4)

Where the coefficients *p* and *q* are determined by the least squares regression with data from four running speeds. An intersection (EOTS; km·h^-1^) between U-shaped quadratic Eq (2) and linear regression line (Eq 4) is obtained when the Eqs 2 and 4 are equal. Rearranging Eqs 2 and 4:

$$av^{2}\;+\;\left(b-p\right)v\;+\;\left(c-q\right)\;=\;0\;.$$ (5)

In Eq 5, *b*-*p* always becomes negative, so that the absolute |*b*-*p*| is regarded as the *b*-*p*. A following formula gives two solutions of Eq 5, and then only a faster one is regarded as the EOTS [7].

$$\text{EOTS }\left(\text{km }\cdot {\text{h}}^{-1}\right)=\;\frac{-(b-p)\pm \;\sqrt{{\left(b-p\right)}^{2}\;-\;4a(c-q)}}{2a}.$$ (6)

### EMG measurement and analysis

After determination of each individual EOTS, EMG recording was conducted during walking and running corresponding to the individual EOTS on every slope at normoxia and hypoxia. The participants were asked to walk and/or run with their freely chosen step frequency for 30 steps. When measuring EMG signals, normoxic conditions were tested prior to hypoxic conditions. This is because muscle activities decreased during high intensity running under hypoxic condition [35], while little information has been available whether muscle activities of the lower leg extremities will be reduced during walking or jogging corresponding to the EOTS under hypoxic condition. The order of slope was randomized at each oxygen condition. Number of analyzed steps was 30.79 ± 0.82 steps (27–34 steps) for all conditions. Its average time durations were 25.605 ± 1.767 seconds for walking and 22.685 ± 1.036 seconds for running, respectively. Each EMG sampling was separated with 1-min standing rest besides the treadmill. Pre-amplified active surface EMG electrode (BA-U410m, Nihon Santeku Co., LTD, Osaka) was placed on *tibialis anterior* (TA), *gastrocnemius medialis* (GM), and *gastrocnemius lateralis* (GL). *Soleus* is a synergist of GM and GL, however, it mainly contributes to the body support, and GM does the forward propulsion regardless of gait pattern or speed [21,27,36–39]. Thus, *soleus* was not measured in our study. Before electrode placement, the skin was shaved and wiped with alcohol for an exfoliation. Electric codes were secured using surgical tape not to disturb locomotive tasks. The EMG signals were amplified with a bio-amplifier (BA 1104B, Digitex Lab Co., LTD, Tokyo). Sampling frequency was set at 2 kHz, and a band-pass filter (8–500 Hz) was applied for the EMG signals. Foot sensor (PS-20KASF4, Kyowa Electronic Instruments Co., LTD., Tokyo) was inserted into a right shoe to count number of steps, and its signal was amplified with a signal conditioner (CDV-700A, Kyowa Electronic Instruments Co., LTD, Tokyo). All signals from each sensor were simultaneously recorded with software (MaP 1038 ver.7.4, Nihon Santeku Co., LTD, Osaka).

At the offline mode, a fast Fourier transform was applied for stored EMG signal to obtain mean power frequency (MPF; Hz). MPF reflects motor unit recruitment in the exercising muscles [40], indicating that an alteration of the MPF values between walking and running at the EOTS can help us to evaluate muscle fiber recruitment pattern in each gait. The sum of the rectified EMG for a particular time duration was used in some previous studies [22,23,25], however, since preferred step frequency could be different between walking and running at the EOTS, the sum of the rectified EMG (μV·sec) was normalized by time duration (sec) and number of steps to cancel the effects of step frequency. This parameter (μV·step^-1^) was regarded as the muscle activity of each muscle.

### Statistical analysis

Data were presented as mean ± SD. A regression analysis using a quadratic equation was applied to the CoT-*v* relationship for walking. A linear regression analysis was applied to the CoT-*v* relationship for running. The CoT values were compared with three-way repeated measures analysis of variance (ANOVA) within participants (FiO_2_ × slope × speed) using online software (ANOVA 4). Two-way repeated measures ANOVA was applied for comparisons of EOTS, ES (FiO_2_ × slope), and EMG data (FiO_2_ × gait). If a significant *F* value was obtained on the dependent variables, Ryan’s *post hoc* test was applied to the appropriate data sets to detect significant mean differences. Its statistical power has been reported to be equivalent to Tukey’s *post hoc* test [41], and it can be used regardless of the data distribution [41]. The statistical significance was set less than 0.05 probability level.

## Results

**Fig 2:**
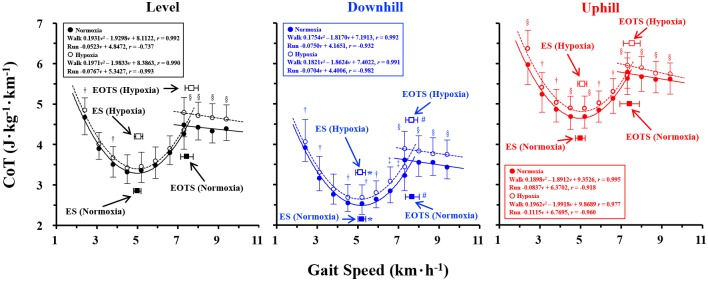
U-shaped (walking) and linear (running) CoT-*v* relationships on different gradient slopes at normoxia and hypoxia. The energetically optimal transition speed (EOTS; km·h^-1^) was 7.447 ± 0.311 km·h^-1^ at normoxia and 7.678 ± 0.324 km·h^-1^ at hypoxia, and the economical speed (ES; km·h^-1^) was 4.993 ± 0.185 km·h^-1^ at normoxia and 5.056 ± 0.210 km·h^-1^ at hypoxia on the level slope (Black panel). The EOTS was 7.679 ± 0.342 km·h^-1^ at normoxia and 7.653 ± 0.295 km·h^-1^ at hypoxia, and 5.198 ± 0.192 km·h^-1^ at normoxia and 5.133 km·h^-1^ ± 0.243 km·h^-1^ at hypoxia on the downhill slope (Blue panel). The EOTS was 7.412 ± 0.480 km·h^-1^ at normoxia and 7.516 ± 0.415 km·h^-1^ at hypoxia, and the ES was 4.984 ± 0.238 km·h^-1^ at normoxia and 5.082 ± 0.223 km·h^-1^ at hypoxia on the uphill slope, respectively. ^#^ downhill > uphill within normoxia or hypoxia. * downhill > level = uphill within normoxia and hypoxia. † (*p* < 0.05), ‡ (*p* < 0.01), and § (*p* < 0.001) indicated significant differences of the energy cost of transport per unit distance (CoT; J·kg^-1^·km^-1^) between normoxia and hypoxia on each slope. Data were shown as mean ± standard deviation (S.D.).

During walking on the uphill and downhill slopes, the CoT values were significantly greater at hypoxia than normoxia at most gait speeds (Fig 2). Significantly greater CoT values at hypoxia were also observed on the level slope, but it was only at slower gait speeds. During running, the CoT values were significantly greater at hypoxia than normoxia at any gait speeds in order of uphill, level, and downhill slopes (Fig 2).

There were no significant differences in the EOTS (*F* = 2.894, *P* = 0.111) and ES (*F* = 1.700, *P* = 0.213) between normoxia and hypoxia on any slopes (Fig 2), while significantly faster EOTS (*F* = 4.619, *P* = 0.019) was observed on the downhill slope at both normoxia and hypoxia. The ES was significantly faster on the downhill slope than level and uphill slopes (*F* = 7.380, *P* = 0.003). Detailed EOTS or ES values were described in Fig 2. Ventilation (*V*_E_; L·min^-1^) was significantly greater at hypoxia than normoxia on any slopes at all running speeds and at most walking speeds (S1 Fig, upper panel). Differences of the *V*_E_ was relatively smaller at faster gait speeds on the level slope (S1 Table), resulting in a non-significant difference at those gait speeds (Fig 2). Arterial oxygen saturation (S_p_O_2_; %) was significantly lower at hypoxia than normoxia, and gradient differences were observed at several gait speeds (S1 Fig, lower panel).

**Fig 3:**
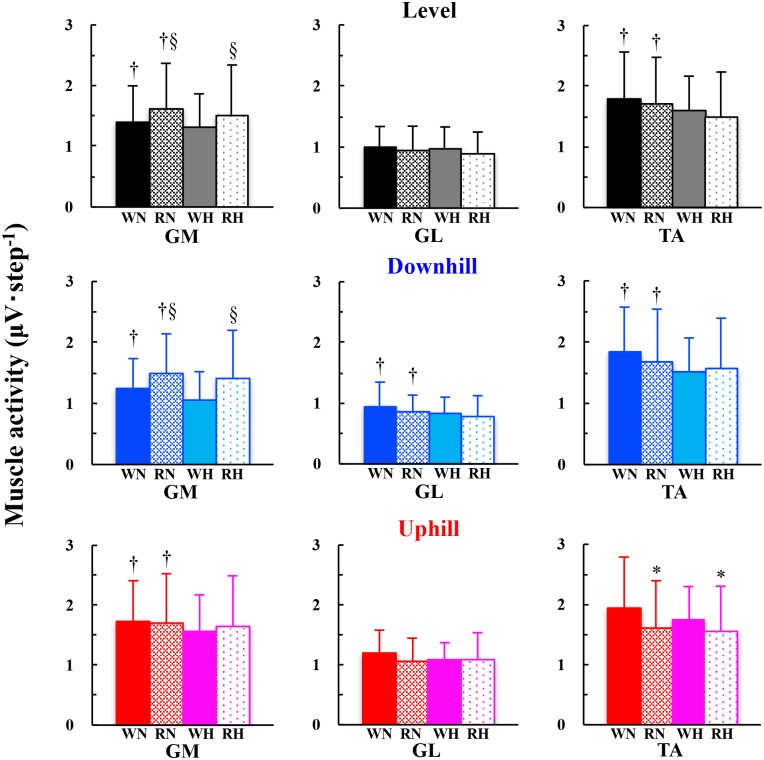
Comparisons of muscle activities during walking or running at the EOTS. Deep colors (black, blue, and red) and thin colors (grey, light blue, and pink) are normoxia and hypoxia, respectively. Solid and dotted bars are walking and running, respectively. WN, RN, WH, and RH mean walking at normoxia, running at normoxia, walking at hypoxia, and running at hypoxia, respectively. * walking > running within normoxia or hypoxia, § walking < running within normoxia or hypoxia, and † normoxia > hypoxia within walking or running, respectively. Data are mean ± S.D.

**Fig 4:**
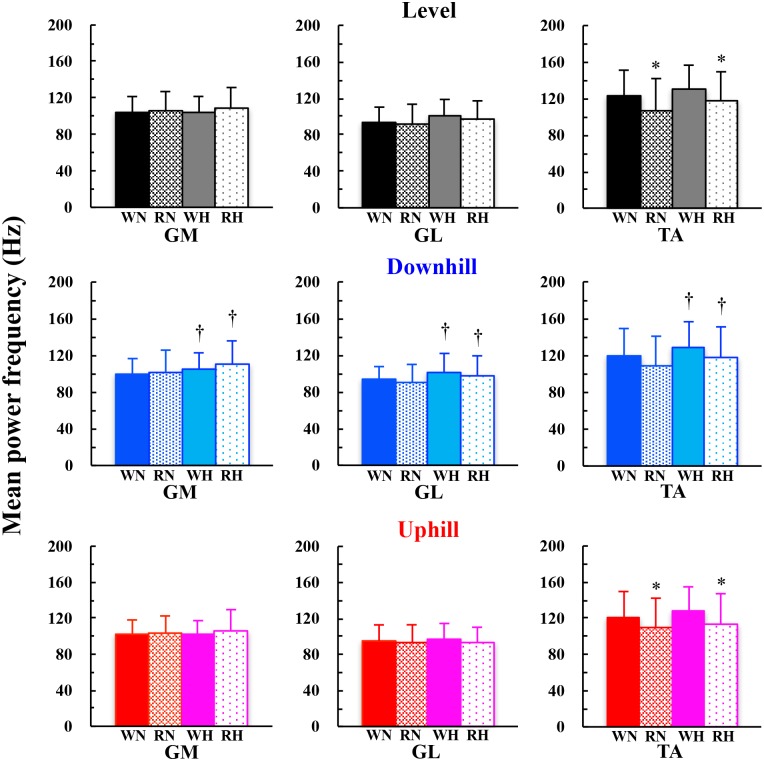
Comparisons of mean power frequency during walking or running at the EOTS. Deep colors (black, blue, and red) and thin colors (grey, light blue, and pink) are normoxia and hypoxia, respectively. Solid and dotted bars are walking and running, respectively. WN, RN, WH, and RH mean walking at normoxia, running at normoxia, walking at hypoxia, and running at hypoxia, respectively. * walk > running within normoxia or hypoxia and † normoxia < hypoxia within walking or running, respectively. Data are mean ± S.D.

Muscle activity of TA became significantly lesser when switching from walking to running at the EOTS on the uphill (*F* = 5.401, *P* = 0.036), but not on the level (*F* = 0.816, *P* = 0.382) and downhill (*F* = 0.135, *P* = 0.718) slopes (* marks in Fig 3). Muscle activity of the GM, one of the antagonists of TA, became significantly greater when running than walking at the EOTS on the level (*F* = 5.468, *P* = 0.035) and downhill (*F* = 7.659, *P* = 0.015) slopes (§ marks in Fig 3). Significantly greater muscle activities were observed at normoxia than hypoxia in the GM on any slopes (*P* = 0.012, 0.010, and 0.005 for downhill, level, and uphill slope, respectively), GL, a synergist of GM and an antagonist of TA, on the downhill slope (*F* = 5.581, *P* = 0.033), and TA on the level (*F* = 6.941, *P* = 0.020) and downhill (*F* = 6.925, *P* = 0.020) slopes († marks in Fig 3). A significantly greater mean power frequency (MPF; Hz) was found in all muscles on the downhill slope at hypoxia than normoxia (*P* = 0.012, 0.029, and 0.035 for GM, GL, and TA, respectively; † marks in Fig 4). MPF of TA was significantly lesser during running than walking on the level (*F* = 5.598, *P* = 0.033) and uphill (*F* = 7.983, *P* = 0.014) slopes (* marks in Fig 4).

## Discussion

### EOTS and ES between normoxia and hypoxia

A significant decrease in the S_p_O_2_ was observed as a function of gait speed (S1 Fig and S1 Table), meaning that our experimental setup successfully designated hypoxic condition. In partly support of our third hypothesis, both EOTS and ES were significantly faster on the downhill slope than the uphill slope under both O_2_ conditions (Fig 2), being in line with a recent result obtained at normoxia [7]. Our study also observed that both EOTS and ES were not significantly different between normoxia and hypoxia on any slopes (Fig 2), while significantly greater CoT values were observed at most gait speeds regardless of gait pattern (Fig 2). Thus, our first hypothesis was rejected. These results indicated that both U-shaped and linear CoT-*v* relationships shifted upward (enhanced CoT values at hypoxia), but not leftward (slower ES), because the ES was unchanged between normoxia and hypoxia (Fig 2). It should be noted that significantly greater CoT values were observed only at slower gait speeds on the level slope (Fig 2). This could be related to relatively smaller percent differences of ventilation between normoxia and hypoxia during walking on the level slope at faster walking speeds (S1 Fig and S1 Table). An upward shift of the U-shaped CoT curve had been reported when walking on the uphill slope [7,13,33,42], pregnant or obese [32,43], or aged [44,45] only at normoxia. Horiuchi et al. [14] recently reported that a significantly slower ES was observed when FiO_2_ was 11%. Such an observation was derived from a linearly increasing trend of the CoT values particularly at faster walking speeds. During uphill locomotion, concentric muscular work, which requires three-fold energy expenditure than eccentric muscular work [46], becomes relatively greater than level and downhill locomotion [47,48]. That is, there is a trend in common with uphill and hypoxia. Insufficient oxygen delivery at hypoxia or higher oxygen demand on the uphill slope could not meet the real metabolic demand at the leg working muscles, indicating that the ES may be affected by the aerobic capacity of each individual.

Most of the recent biomechanical studies employed PTS, but not EOTS. Note that a series of metabolic measurements are not necessary when using the PTS. There are ‘several’ criteria for determining the PTS [49], suggesting that the PTS could appear by multiple factors. Here, it is questionable whether the biomechanically determined PTS is comparable to the metabolically determined EOTS. Indeed, the EOTS coincided with the PTS in well-trained racewalkers [50], although it was slightly faster than the PTS in young active males [6,8,10,18] and distance runners [9]. In either case, both EOTS and PTS appeared around 7–8 km h^-1^ in those previous studies. In contrast to our proposed ‘shifting model’ of the U-shaped and linear CoT-*v* relationships during human bipedal locomotion, our results provided only an upward shift (Fig 1b), but not a combination of upward and leftward shifts (Fig 1a), of both U-shaped and linear CoT-*v* relationships at moderate hypoxia on the level and gradient slopes, resulting in a non-significant change in the EOTS and ES.

### Muscle activity at the EOTS

In partly support of our second hypothesis, a significantly lesser TA activity was observed when switching gait pattern from walking to running at the EOTS on the uphill slope (* marks in Fig 3), but not on the level and downhill slopes (Fig 3). In our study, EMG was sampled during walking and running at the EOTS, but not at the PTS. However, our results were not necessarily in agreement with some related previous studies [22–27]. Note that there is a methodological difference when evaluating muscle activities. The sum of the rectified EMG for a particular time duration was used in some previous studies [22,23,25]. However, preferred step frequency could be different between walking and running at the EOTS, so that each rectified EMG should be normalized not only by time duration but also by number of steps (μV·step^-1^) to control for the effects of number of steps. Instead, a significant decrease in the MPF was observed in the TA when switching gait pattern from walking to running on the level and uphill slopes (Fig 4). These observations indicated that muscle fiber recruitment pattern shifted from fast (Type II) to slow (Type I) twitch fibers, because MPF reflects motor unit action potential [40,51]. Thus, a significant decrease in the MPF of the TA when switching gait pattern on the level and uphill slopes (Fig 4) could reflect that more slow twitch fibers are recruited during running than walking at the EOTS. Indeed, muscle fiber composition of the TA in humans is rich in slow fibers in both superficial and deep area [52,53]. Shih et al. [54] observed the PTS at 7.33 km·h^-1^, and they found that only TA activity was greater during walking than running at that speed. These previous findings suggest that the TA must be the most sensitive for the gait change in erect bipedalism. If more slow twitch fibers are recruited without an increase in muscle activities during running than walking at the EOTS, then it will contribute to reduce metabolic cost, resulting in avoiding an early onset of localized muscle fatigue in the TA.

Other muscle activities in ankle plantar flexors, such as *gastrocnemius medialis* (GM) and *gastrocnemius lateralis* (GL), should be also focused, because these two muscles are the antagonists of the TA. In fact, ankle plantar flexors play an essential role in generating mechanical power output during human locomotion [21,27,36–39,55], although combined information between these muscle activities and whole-body metabolic cost during either walking or running at the EOTS under hypoxic condition has not been available yet. In our present study, significantly greater GM activities were observed when running than walking at the EOTS on the level and downhill slopes (Fig 3). It is apparent that a significant increase in the GM activity does not contribute to minimize the cost of muscle activities. Muscular activities of both planter flexors gradually increased in an order of downhill, level, and uphill slopes (Fig 3). As noted before, a significant increase in the planter flexors was observed only in the GM, but not in the GL, on the level and downhill slopes (Fig 3). These results indicated that the GM was more responsible for accelerating the body during running on the level and gentle downhill slopes. Note that the TA activity significantly decreased only on the uphill slope (Fig 3). This unique behavior of planterflexors and dorsiflexor might be related to a favor of more fore-foot strikers in the present study, because habitual forefoot strikers are characterized by larger peak planter flexion moments [56,57] with a concomitant lesser TA activity [58]. It was also reported that an increase in the plantar flexor activity might serve as another trigger for gait change through afferent information to the locomotor central pattern generators [59]. Such explanations could be related to kinematic changes between walking and running near the gait transition speed [21,49,60], because different joint angle and angular velocity generate different muscle activation patterns. Minimization of the muscle activities is particularly necessary to minimize the metabolic cost for avoiding early onset of localized muscle fatigue. A biological benefit of such a gradual shift of muscle activation pattern is a ‘selectability’ of either walking or running around the EOTS in humans.

During high-intensity dynamic exercise at hypoxia, such as repeated bouts of short sprint [35,61] and exhaustive cycling [62,63], locomotor muscle fatigue was accelerated due to a reduced arterial oxygenation. We observed that S_p_O_2_ ranged from 80% to 90% at hypoxia (S1 Fig), being equivalent to the results obtained in some previous studies [35,62,63]. In our study, most muscle activities became lesser at hypoxia than normoxia († marks in Fig 3; GM on any slopes, GL on the downhill slope, and TA on the level and downhill slopes), while significantly greater MPF was observed at hypoxia than normoxia in all muscles on the downhill slope regardless of gait pattern († marks in Fig 4). In other words, MPF was not significantly lesser at hypoxia than normoxia in all muscles (Fig 4). These results clearly evidenced that the locomotor muscle fatigue did not occur in any muscles even at hypoxia, because muscle fatigue during sustained force output has been known when MPF was decreased with an increased muscle activity [40]. A greater MPF in any muscles on the downhill slope indicated that more fast twitch fibers were recruited at hypoxia on that slope. Note that any muscle activities were not increased at hypoxia than normoxia (Fig 3). Such a trend was independent of gait pattern (Figs 3 and 4). A shift of the muscle recruitment pattern toward fast twitch fibers could be more advantageous rather than an increase in the muscle activities for minimizing the whole body metabolic cost.

## Conclusions

Both ES and EOTS were not significantly different between normoxia and hypoxia on any slopes (Fig 2). GM, but not GL, activated more when the gait pattern was changed from walking to running on the level and gentle downhill slopes (Fig 3). Significant decreases in the muscular activity and/or MPF when switching the gait pattern were observed only in the TA (Figs 3 and 4). Given these results, neither the EOTS nor the ES is slowed by moderate hypoxia in the population of this study. Muscular activities of the lower leg extremities in association with those muscle fiber recruitment patterns are dependent on the gradient difference when walking or running at the EOTS.

## Supporting information

S1 Fig Ventilatory responses during walking and running at normoxia and hypoxia. In the upper panel, black, blue, and red colors represent level, downhill, and uphill slope, respectively. Solid plots and lines are normoxia. Open plots and dotted lines are hypoxia. Minute ventilation (*V*_E_; L·min^-1^) was significantly higher at hypoxia than normoxia during running on ant slopes (*p* < 0.01). In contrast, there were several gait speeds which the *V*_E_ was not significantly higher at hypoxia than normoxia during walking, so that statistical, absolute, and percent differences during walking are summarized in S1 Table. Lower panel clearly showed significant differences in the arterial oxygen saturation between normoxia and hypoxia. Each plot and color are the same as the upper panel. * Normoxia > Hypoxia and Downhill < Level < Uphill at any speeds. Data are mean ± S.D.
(TIF)

S1 Table Absolute and percent differences of minute ventilation (*V*_E_) during walking between normoxia and hypoxia. Values are presented as absolute (L·min^-1^) and percent difference of *V*_E_ between normoxia and hypoxia. Bold letters indicate significantly greater *V*_E_ at hypoxia than normoxia.
(PDF)
