# Supplementary figures and images for: Muscle activities during walking and running at energetically optimal transition speed under normobaric hypoxia on gradient slopes
Source: PLoS One. 2017 Mar 16;12(3):e0173816. doi: 10.1371/journal.pone.0173816 (PMC5354415; doi:10.1371/journal.pone.0173816)

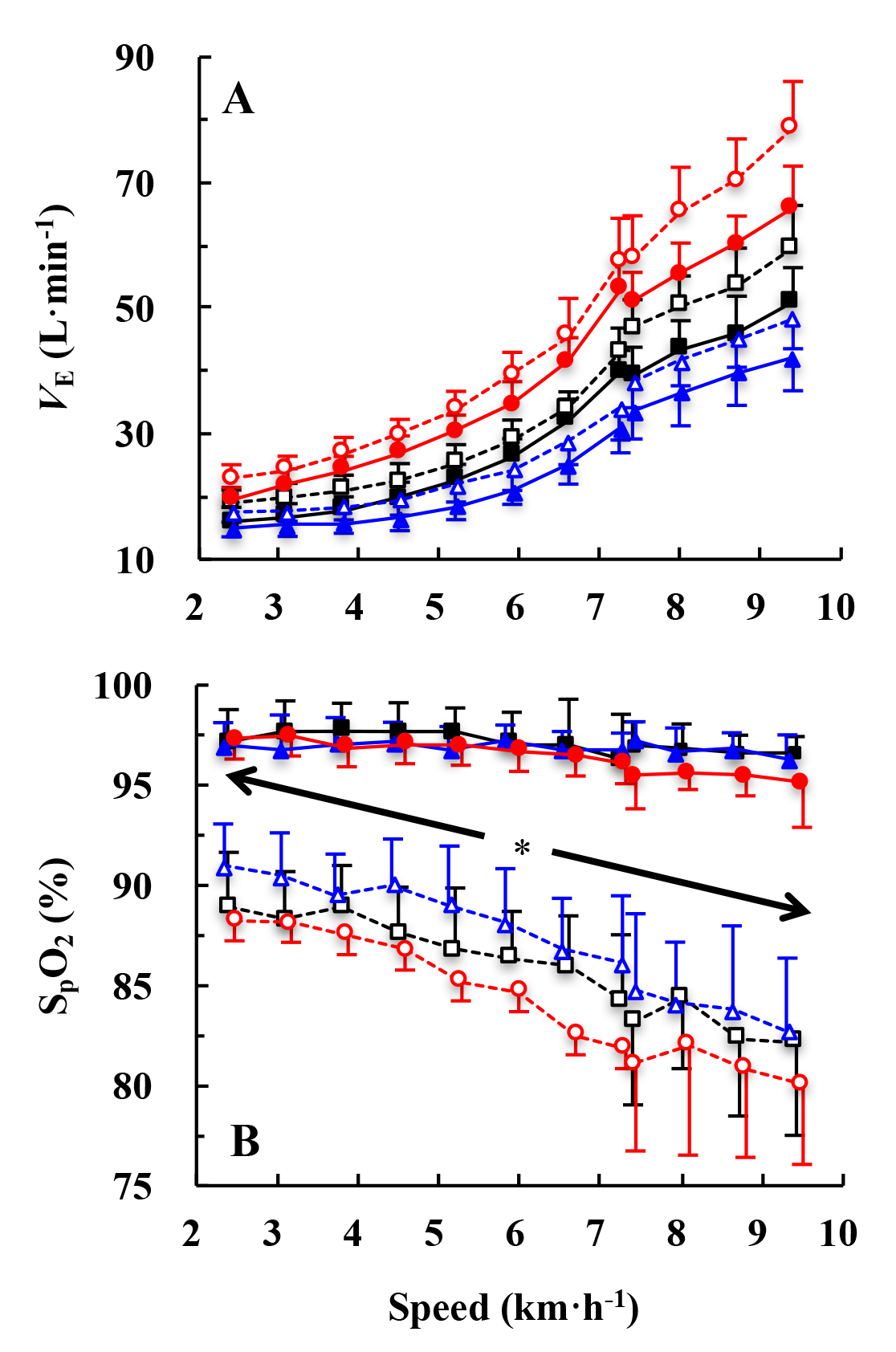

Supplement: S1 Fig — In the upper panel, black, blue, and red colors represent level, downhill, and uphill slope, respectively. Solid plots and lines are normoxia. Open plots and dotted lines are hypoxia. Minute ventilation (VE; L·min-1) was significantly higher at hypoxia than normoxia during running on ant slopes (p < 0.01). In contrast, there were several gait speeds which the VE was not significantly higher at hypoxia than normoxia during walking, so that statistical, absolute, and percent differences during walking are summarized in S1 Table. Lower panel clearly showed significant differences in the arterial oxygen saturation between normoxia and hypoxia. Each plot and color are the same as the upper panel. * Normoxia > Hypoxia and Downhill < Level < Uphill at any speeds. Data are mean ± S.D. (TIF) [file pone.0173816.s001.tif]
