# Supplementary material for: Muscle activities during walking and running at energetically optimal transition speed under normobaric hypoxia on gradient slopes
Source: PLoS One. 2017 Mar 16;12(3):e0173816. doi: 10.1371/journal.pone.0173816 (PMC5354415; doi:10.1371/journal.pone.0173816)
Supplement: S1 Table — Values are presented as absolute (L·min-1) and percent difference of VE between normoxia and hypoxia. Bold letters indicate significantly greater VE at hypoxia than normoxia. (PDF) [file pone.0173816.s002.pdf]

Speed (km·h^{-1})	2.4	3.1	3.8	4.5	5.2	5.9	6.6	7.3
Level	**2.98 (18.6%)**	2.80 (15.3%)	**3.34 (19.2%)**	2.76 (13.9%)	2.75 (10.7%)	2.79 (9.4%)	1.82 (7.7%)	3.16 (6.4%)
Downhill	**2.58 (16.2%)**	**2.25 (13.5%)**	2.72 (14.6%)	**2.92 (15.3%)**	**3.41 (16.6%)**	**3.39 (15.5%)**	**3.75 (12.1%)**	**3.25 (10.1%)**
Uphill	**3.49 (18.0%)**	**2.43 (11.6%)**	**2.72 (12.3%)**	**2.94 (12.4%)**	**3.44 (11.5%)**	**4.85 (13.8%)**	**4.00 (10.3%)**	4.51 (8.2%)
